# Defective Lamtor5 Leads to Autoimmunity by Deregulating v‐ATPase and Lysosomal Acidification

**DOI:** 10.1002/advs.202400446

**Published:** 2024-04-19

**Authors:** Wei Zhang, Zhou Sha, Yunzhe Tang, Cuiyuan Jin, Wenhua Gao, Changmai Chen, Lang Yu, Nianyin Lv, Shijia Liu, Feng Xu, Dandan Wang, Liyun Shi

**Affiliations:** ^1^ School of Medicine Nanjing University of Chinese Medicine Nanjing 210046 China; ^2^ Key lab of Artificial Organs and Computational Medicine Institute of Translational Medicine Zhejiang Shuren University Hangzhou 310022 China; ^3^ School of Pharmacy Fujian Medical University Fuzhou 350122 China; ^4^ The Affiliated Hospital of Nanjing University of Chinese Medicine Nanjing 210029 China; ^5^ Department of Infectious Diseases The Second Affiliated Hospital Zhejiang University School of Medicine Hangzhou 310009 China; ^6^ Department of Rheumatology and Immunology The Affiliated Drum Tower Hospital of Nanjing University Medical School Nanjing 210093 China

**Keywords:** autoimmunity, lamtor5, lysosome, v‐ATPase

## Abstract

Despite accumulating evidence linking defective lysosome function with autoimmune diseases, how the catabolic machinery is regulated to maintain immune homeostasis remains unknown. Late endosomal/lysosomal adaptor, MAPK and mTOR activator 5 (Lamtor5) is a subunit of the Ragulator mediating mechanistic target of rapamycin complex 1 (mTORC1) activation in response to amino acids, but its action mode and physiological role are still unclear. Here it is demonstrated that Lamtor5 level is markedly decreased in peripheral blood mononuclear cells (PBMCs) of patients with systemic lupus erythematosus (SLE). In parallel, the mice with myeloid Lamtor5 ablation developed SLE‐like manifestation. Impaired lysosomal function and aberrant activation of mTORC1 are evidenced in Lamtor5 deficient macrophages and PBMCs of SLE patients, accompanied by blunted autolysosomal pathway and undesirable inflammatory responses. Mechanistically, it is shown that Lamtor5 is physically associated with ATP6V1A, an essential subunit of vacuolar H^+^‐ATPase (v‐ATPase), and promoted the V0/V1 holoenzyme assembly to facilitate lysosome acidification. The binding of Lamtor5 to v‐ATPase affected the lysosomal tethering of Rag GTPase and weakened its interaction with mTORC1 for activation. Overall, Lamtor5 is identified as a critical factor for immune homeostasis by intergrading v‐ATPase activity, lysosome function, and mTOR pathway. The findings provide a potential therapeutic target for SLE and/or other autoimmune diseases.

## Graphical abstract

In this article, it is demonstrated that late endosomal/lysosomal adaptor, MAPK and mTOR activator 5 (Lamtor5) is physically associated with vacuolar H^+^‐ATPase (v‐ATPase) subunit ATP6V1A to promote lysosomal acidification, and simultaneously affected the Rag GTPase/mTORC1 interaction at lysosomes. This study established Lamtor5 as a critical regulator for immune homeostasis via regulating lysosomal v‐ATPase and mTORC1 pathway, providing a novel insight into the pathogenesis of autoimmune diseases.

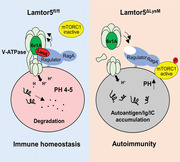

## 1 Introduction

The immune system at steady state is regulated by a fine‐tuning mechanism integrating multiple signaling pathways and most of the pathways are converged at lysosomes. Lysosomes are a major degradative system with the ability to eradicate cellular debris/aggregates, and also it is a central signaling hub orchestrating a wide range of vital biological activities.^[^ ^1^, ^2^, ^3^ ^]^ By eliminating cellular wastes through the endocytic and autophagic pathways, lysosomes play a key role in controlling cellular quality and tissue homeostasis. Impaired lysosomal clearing function or blunted autolysosomal pathway cause the accumulation of apoptotic debris and/or immune complexes, leading to aberrant activation of immune cells and inflammatory signaling and even autoimmune diseases.^[^ ^4^, ^5^ ^]^

The catabolic activity of lysosomes, the last step of the autophagic flux for waste disposal, is executed by a network of hydrolytic enzymes such as proteases, glycosidases, and lipases. The hydrolyses are generally inactive unless they are processed under highly acidic pH within a lysosomal lumen, and lysosomal acidity is essentially governed by vacuolar H^+^‐ATPase (v‐ATPase). The v‐ATPase is a universal proton pump composed of a peripheral V1 domain specialized in ATP hydrolysis, and a membrane integral V0 domain responsible for proton pumping and lysosome acidification.^[^ ^6^, ^7^ ^]^ Studies have shown that the cytosolic V1 subunit of v‐ATPase is activated upon ligation with the V0 component, which would subsequently induce the energy transferred to the V0 subunit and promote protons pumping for lysosomal acidification.^[^ ^8^, ^9^ ^]^ Dysfunctional v‐ATPase and de‐acidic lysosomes have been involved in the pathogenesis of degenerative and autoinflammatory diseases including lupus‐like autoimmunity.^[^ ^10^, ^11^ ^]^ Therefore, intensive efforts are currently being made to elucidate the mechanism regulating v‐ATPase activity and lysosomal integrity. A number of new molecules, such as serine/threonine kinase 11 interacting protein (STK11IP), N‐deacetylase and N‐sulfotransferase 3 (NDST3), and Ubiquilins (UBQLNs), have been recently discovered to regulate v‐ATPase and lysosomal function.^[^ ^12^, ^13^, ^14^ ^]^ However, the knowledge about the v‐ATPase regulation and its relevance to inflammatory signaling is far from sufficient, and new regulators need to be identified.

On the other hand, the lysosome functions as a signaling nexus with a host of signaling molecules converging onto it, and the mechanistic target of rapamycin complex 1 (mTORC1) plays a central role in regulating lysosome activity and degradation. mTORC1 is a lysosome‐residing Ser/Thr kinase capable of sensing nutritional, hormonal, and energetic cues to translate them into cellular physiology.^[^ ^15^, ^16^ ^]^ The activity of mTORC1 is dynamically regulated by a signaling cascade involving Ragulator and Rag GTPase. The Ragulator, also known as lysosomal adaptor and MAPK and mTOR activator/regulator (LAMTOR), has long been regarded as a scaffolding protein and a guanine nucleotide exchange factor (GEF) for mTORC1 activation via association with Rag GTPase. Generally regarded as an anchor for Rag and mTORC1 at the lysosome, the Ragulator components (Lamtor1‐5), however, were recently found to have distinct and vital biological functions.^[^ ^17^, ^18^, ^19^ ^]^ Lamtor5 is a newly identified member of the Ragulator that is preferentially localized at cellular membrane systems including lysosome surface.^[^ ^20^ ^]^ Lamtor5 is originally recognized as an oncogenic protein with the ability to promote cellular proliferation and survival,^[^ ^21^, ^22^, ^23^ ^]^ but recent data indicate that Lamor5 may have non‐carcinogenic roles such as the immunoregulatory function.^[^ ^24^ ^]^ Global deletion of Lamtor5 caused embryonic lethality in mice,^[^ ^25^ ^]^ implying a crucial and non‐redundant role for this molecule, of which we know little. Another critical but poorly understood question is whether the Ragulator component like Lamtor5 would interact with v‐ATPase and/or Rag GTPase to modulate mTORC1 activity. Accumulating evidences have indicated that well‐organized mTOR activity is critical for immune homeostasis and preventing autoimmunity, but how the lysosomal v‐ATPase, Ragulator, and mTORC1 are integrated to control myeloid cell function and immune responses is yet to be explored.^[^ ^26^, ^27^ ^]^

In this study, we demonstrate that defective expression of Lamtor5, accompanied by impaired lysosome activity and hyperactivation of mTORC1, was evidenced in SLE patients. In accordance, myeloid Lamtor5 ablating mice (Lamtor5^ΔLysM^) developed progressive inflammatory pathology and SLE‐like autoimmunity. Further studies revealed that Lamtor5 physically associated with v‐ATPase subunit (ATP6V1A) to promote lysosomal acidification, and simultaneously affected the Rag/mTORC1 interaction at lysosomes. We thus established Lamtor5 as a critical regulator for immune homeostasis via regulating lysosomal v‐ATPase and mTORC1 pathway, providing a novel insight into the pathogenesis of autoimmune diseases.

## 2 Results

### 2.1 Defective Lamtor5 Expression is Associated with SLE Pathogenesis in Human and Mice

**Figure 1:**
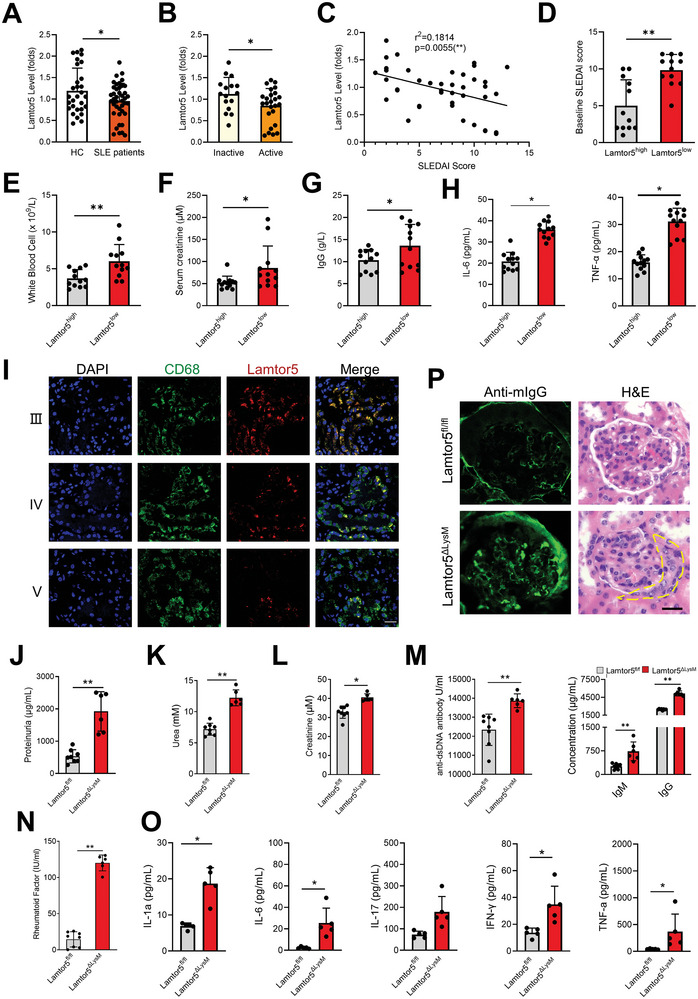
The expression of Lamtor5 in myeloid cells correlates with the severity of SLE. A) Lamtor5 levels in PBMCs from lupus patients (n = 41) and HC (healthy control, n = 29) were analyzed by qPCR. B) Lamtor5 level in PBMCs of active SLE patients (SLEDAI≥6, n = 25) and inactive SLE patients (SLEDAI<6, n = 16). C) Correlation between Lamtor5 levels and SLE Disease Activity Index (SLEDAI) scores in SLE patients. R, spearman correlation coefficient. SLEDAI scores D), amounts of white blood cells E), serum levels of creatinine F), IgG G), and proinflammatory cytokine H) in SLE patients with a high or low level of Lamtor5. I) Representative images of immunofluorescence staining of Lamtor5 (red) and macrophage marker CD68 (green) in kidney biopsy sections of patients with lupus nephritis LN (Class III, IV, V). Nuclei, DAPI (blue). Scale bar, 10 µm. J–N) The concentrations of proteinuria, serum urea, creatinine, serum levels of anti‐dsDNA autoantibody, IgM, IgG, and rheumatoid factor detected in samples from 4‐month‐old Lamtor5^fl/fl^ (n = 8) and Lamtor5^ΔLysM^ (n = 6) mice. O) The Bio‐plex analysis of the serum level of the indicated cytokines from samples of 4‐month‐old Lamtor5^fl/fl^ (n = 5) and Lamtor5^ΔLysM^ (n = 5) mice. P) Immunofluorescence staining (Alexa Fluor 488‐conjugated anti‐mouse IgG), or H&E staining of kidney sections from 8‐month‐old Lamtor5^fl/fl^ or Lamtor5^ΔLysM^ mice. The dotted area represents the crescentic lesion. Scale bar, 20 µm. All results are expressed as the mean ± SD. ^*^ *P* <0.05, ^**^ *P* <0.01 by Student's *t* test.

By analyzing peripheral blood mononuclear cells (PBMCs) from gender and age‐matched SLE subjects (n = 41) and healthy individuals (n = 29) (Table S1, Supporting Information), we found that the level of Lamtor5 was significantly decreased in SLE patients compared with that in healthy controls (**Figure** **1**A). A much lower level of Lamtor5 was detected in PBMCs of active SLE patients (SLE disease activity index [SLEDAI]≥6, n = 25), as compared with that in subjects with inactive SLE (SLEDAI <6, n = 16) (Figure 1B). The correlation analysis confirmed that the Lamtor5 level was negatively correlated with disease scores (Figure 1C). Among patients with SLE, those with a Lamtor5 level at the bottom 30% (Lamtor5^low^, n = 12), exhibited higher SLEDAI scores than those with a Lamtor5 level at the top 30% (Lamtor5^high^, n = 12). Concurrently, the hallmarks of autoimmunity, including counts of white blood cells, serum levels of creatinine and IgG, and production of proinflammatory cytokines including TNF‐α and IL‐6 were significantly elevated in Lamtor5^low^ patients relative to Lamtor5^high^ subjects (Figure 1D**–**H). Moreover, the expression of Lamtor5 and its colocalization with CD68^+^ macrophages decreased significantly in renal biopsy samples from patients with lupus nephritis (LN), especially in class IV and V (Figure 1I). The data thus indicated that deregulated Lamtor5, in macrophages particularly, was involved in the pathogenesis of SLE pathology in humans.

To further evaluate the functional importance of Lamtor5 in SLE pathogenesis, we constructed the mice with conditional knockout (cKO) of Lamtor5 in myeloid cell lineage (Lamtor5^ΔLysM^) (Figure S1A–D, Supporting Information). Initial studies demonstrated that Lamtor5^ΔLysM^ mice, compared with age‐ and sex‐matched Lamtor5^fl/fl^ mice, displayed no evident changes in the appearance, behavior, and body weights at an early stage after birth. However, levels of urine protein, serum urea, creatinine, and rheumatoid factor, the tiers of anti‐dsDNA antibodies and IgM/G levels, as well as the serum levels of proinflammatory cytokines were significantly elevated in Lamtor5 ablating mice (Figure 1J–O). The pathological changes in the kidneys however were insignificant in Lamtor5^ΔLysM^ mice at the age of 4 months (Figure S1E, Supporting Information). Considering that cytokine changes might precede symptoms manifestation, we further examined 8‐month‐old Lamtor5^ΔLysM^ females and found that the animals exhibited glomerulonephritis‐like pathology, characterized by an expanded double layer around the glomeruli, thicker tubules, glomerular hypercellularity and glomerular IgG deposition (Figure 1P), which was reminiscent of SLE‐like manifestation in human (Figure 1D–I). Together, the data showed that mice with myeloid deletion of Lamtor5 developed SLE‐like pathologies with evident glomerulonephritis, autoantibodies production, and spontaneous inflammation.

### 2.2 Lamtor5 Deficient Macrophages Display Hyperactivated and Inflammatory Phenotypes

**Figure 2:**
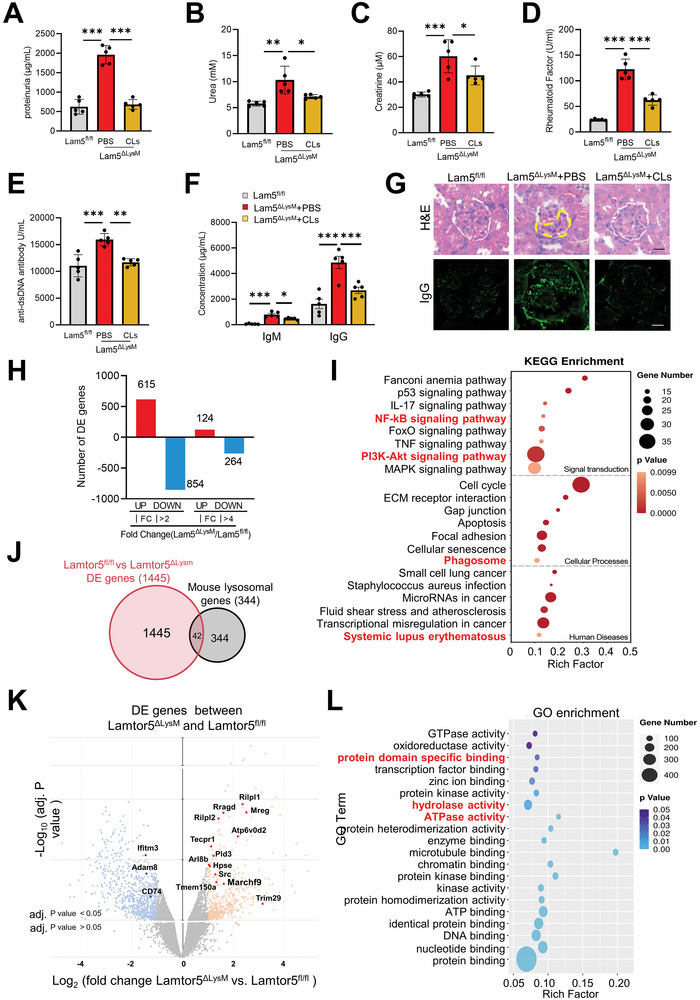
Lamtor5 deficient macrophages display hyperactive and inflammatory phenotype. A–G) Macrophage depletion ameliorates the lupus‐like phenotype in Lamtor5^ΔLysM^ mice. Age‐matched female Lamtor5^ΔLysM^ mice (n = 5) were treated with either vehicle or clodronate liposomes (CLs, 100 µg mouse^−1^) twice a week for 1 month starting at 8 months of age. Lamtor5^fl/fl^ mice were used as controls. The concentrations of proteinuria, serum urea, creatinine, rheumatoid factor, serum levels of anti‐dsDNA autoantibody, IgG and IgM were determined. Immunofluorescence staining (Alexa Fluor 488‐conjugated anti‐mouse IgG), or H&E staining of kidney sections. Dotted area represents the crescentic lesion. Scale bar, 20 µm. H–L) Transcriptomics analysis of BMDMs from 4‐month‐old Lamtor5^ΔLysM^ and Lamtor5^fl/fl^ mice (n = 3). Differentially expressed (DE) genes with fold change (FC) differences at *P* < 0.05; KEGG pathways analysis of DE genes; Overlap between DE genes identified by RNA‐seq and mouse lysosomal genes; Volcano plot of DE genes associated with lysosomes. Blue dots, downregulated; red dots, upregulated; Gene ontology (GO) analysis of function of DE genes. All results are expressed as the mean ± SD. ^*^ *P* < 0.05, ^**^ *P* < 0.01, ^***^ *P* <0.001 by Student's *t* test.

Since deregulated macrophage has been critically involved in the pathogenesis of autoimmune diseases including SLE,^[^ ^28^, ^29^, ^30^ ^]^ we next explored the impact of Lamtor5 on macrophage phenotype and function. The results showed that the expression of macrophage surface markers like MHCII and costimulatory molecules CD80, CD86, and CD40 levels were upregulated in Lamtor5 ablating macrophages relative to control cells (Figure S2A, Supporting Information). Meanwhile, knocking out Lamtor5 in macrophages also enhanced its phagocytic function significantly (Figure S2B, Supporting Information). The levels of proinflammatory cytokines and key M1 marker genes were increased, whereas that of the immunosuppressive cytokines and M2‐featured genes were repressed in Lamtor5 ablating macrophages (Figure S2C,D, Supporting Information). The data indicated that Lamtor5 lacking macrophages underwent a hyperactivated and inflammatory phenotype shift, in line with the proinflammatory phenotype of PBMCs observed in SLE patients. To confirm that the lupus‐like phenotype associated with Lamtor5 loss was mainly caused by deregulated macrophages, we next depleted macrophages in Lamtor5^ΔLysM^ mice by clodronate liposomes (CL). Remarkably, the data showed that macrophage depletion significantly alleviated SLE‐like pathologies in Lamtor5^ΔLysM^ mice, as evidenced by lower levels of urine protein, serum urea, creatinine, and rheumatoid factor, deceased tiers of anti‐dsDNA antibodies and IgM/G levels, as well as improved renal pathological changes (**Figure** **2**A **–**G). The data thus indicated that macrophages with Lamtor5 ablation played a dominant role in SLE pathogenesis.

Next, to further understand the genomic profile of Lamtor5 ablation macrophages, we conducted the transcriptome analysis by RNA‐sequencing (RNA‐seq) on bone marrow‐derived macrophages (BMDMs) from 4‐month‐old Lamtor5^fl/fl^ or Lamtor5^ΔLysM^ mice. The results showed that 124 genes were upregulated and 264 genes (fold change >4 and *P* <0.05) were downregulated in Lamtor5 knockout (KO) macrophages relative to control cells (Figure 2H). Kyoto Encyclopedia of Genes and Genomes (KEGG)‐based analysis revealed that the enriched differentially expressed (DE) genes in Lamtor5^−/−^ versus Lamtor5^+/+^ macrophages were related to cellular proliferation, senescence, inflammation, phagolysosome and autoimmunity (e.g., PI3K‐Akt, NF‐κB, phagosome, and SLE) (Figure 2I). Considering that autoinflammation is frequently associated with blocked autolysosomal pathways and impaired cellular degradative machinery, we further compared the differentially expressed (DE) genes with lysosomal genes based on the Mouse Lysosomal Gene Database (http://lysosome.unipg.it/mouse.php). The comparative analysis identified that 42 lysosome‐associated genes, such as Rilpl2, Atp6v0d2, TMEM150a, and IFITM3, were differentially expressed in Lamtor5 KO macrophages relative to their counterparts (Figure 2J,K). Consistently, the Gene Ontology (GO) analysis revealed the enrichment of lysosome‐related genes including hydrolase, ATPase, and ATP binding, in Lamtor5 lacking macrophages (Figure 2L). In support, we observed that PBMCs of SLE patients expressed defective expression of lysosomal genes relative to healthy controls (Figure S2E, Supporting Information). The data thus indicated that loss of Lamtor5 rendered macrophages shift to spontaneously activated and inflammatory phenotypes, which was likely associated with impaired lysosomal function.

### 2.3 Lamtor5 Ablation Causes Lysosome De‐Acidification and Blocked Autophagic Flux

**Figure 3:**
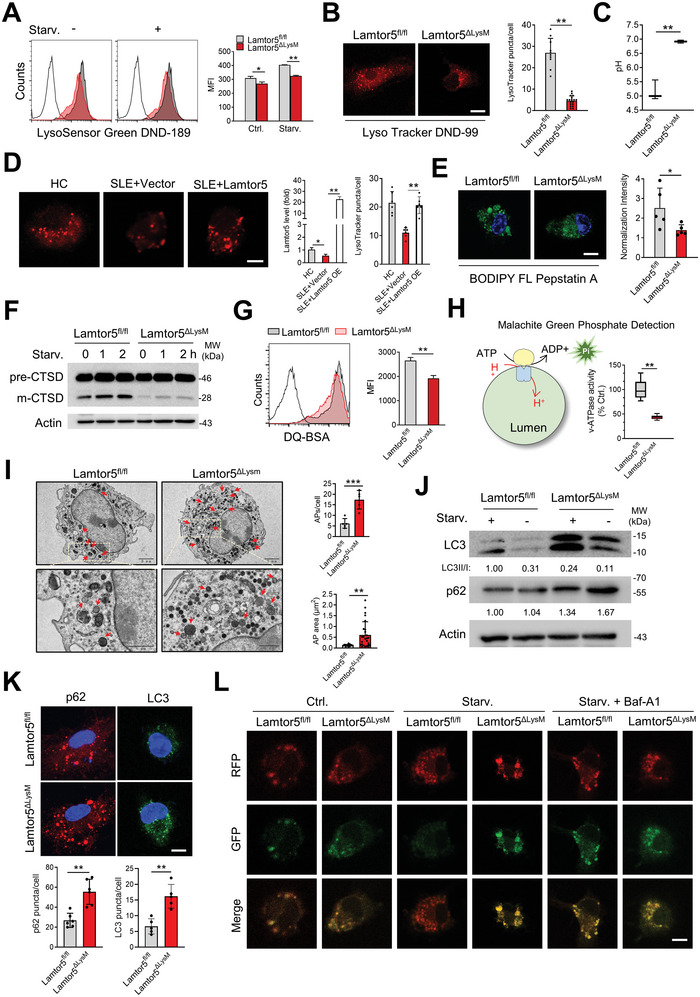
Loss of Lamtor5 impairs lysosomal acidification and autophagic flux in macrophages. BMDMs from 4‐month‐old Lamtor5^ΔLysM^ and Lamtor5^fl/fl^ mice were prepared for functional analysis. A) FACS analysis of LysoSensor Green DND‐189 staining. B) Confocal microscopy of LysoTracker DND‐99 staining. Scale bar, 5 µm. C) Lysosomal pH was measured using LysoSensor Yellow/Blue DND‐160. D) PBMCs of SLE patients were transfected with Lamtor5‐expressing or control plasmids (pCMV‐tag2B‐Lamtor5 or pCMV‐tag2B) using lipofectamine 2000 for 48 h. The expression of Lamtor5 was determined by Q‐PCR and lysosomal acidification was determined using LysoTracker DND‐99 staining. Scale bar, 5 µm. E) BODIPY FL‐pepstatin A staining. Scale bar, 5 µm. F) Immunoblot analysis of CTSD in macrophages upon serum starving for indicated time periods. G) Flow cytometry of DQ‐BSA degradation. H) Determination of v‐ATPase activity. I) TEM of macrophages showing the accumulation of autophagosomes (red arrow). The amounts and areas of autophagosomes per cell were quantified. Scale bars, 2 µm (upper) and 1 µm (lower). J) Immunoblotting for LC3 and p62 in macrophages cultured with or without FBS for 2 h. K) Immunofluorescence staining of p62 and LC3 puncta. Scale bar, 5 µm. L) Confocal microscopy of macrophages that were transfected with mCherry‐EGFP‐LC3B‐expressing plasmids, followed by Baf‐A1 treatment (50 µm for 6 h) or not and FBS deprivation for 24 h. Scale bar, 5 µm. All results are expressed as the mean ± SD. ^*^ *P* < 0.05, ^**^ *P* < 0.01, ^***^ *P* <0.001 by Student's *t* test.

Given the above findings, we set out to determine whether and how Lamtor5 affected lysosomal function in macrophages. Considering that the acidic luminal environment was required for lysosomal hydrolytic enzymes and catabolic activity, we therefore assessed the acidic status of lysosomes. Remarkably, Lamtor5 KO macrophages exhibited lower fluorescence intensity of LysoSensor Green and LysoTracker Red, indicative of impaired lysosome acidity (**Figure** **3**A,B). Using LysoSensor Yellow/Blue DND‐160, a dual‐emission ratiometric probe, we demonstrated that Lamtor5 KO macrophages displayed greatly heightened lysosomal pH compared with their counterparts (Figure 3C). Thus, the data consistently demonstrated that loss of Lamtor5 affected lysosomal acidification and elevated pH in macrophages. As additional support, we showed that PBMCs from SLE patients were featured with impaired lysosomal acidification, and more importantly, enforced expression of Lamtor5, to a great extent, resumed lysosomal acidity in these cells (Figure 3D).

Next, we assessed the activity of hydrolytic enzymes as they are indicative of lysosomal function and also critical for lysosome degradative activity. The result showed that Lamtor5 ablating macrophages exhibited significantly reduced BODIPY FL‐pepstatin A staining, a dye probe specifically binding active CTSD (Figure 3E). The data indicated compromised lysosomal catabolism upon Lamtor5 loss in macrophages. In parallel, cleavage of CTSD induced by serum‐starving, suggestive of lysosomal acidity, was found to be compromised in Lamtor5 KO macrophages (Figure 3F). Using self‐quenched fluorophore DQ‐BSA, we also revealed that Lamtor5 KO macrophages elicited blunted lysosomal acidity much compared with their counterparts (Figure 3G). Together, the results consistently demonstrated that Lamtor5 ablation caused impaired lysosomal acidification and blunted degradative capability in macrophages.

As is known, lysosomal acidic pH is essentially governed by the activity of vacuolar‐type H⁺ ATPase (v‐ATPase), a well‐characterized proton pump. We therefore further tested the impact of Lamtor5 on lysosomal v‐ATPase activity by exploiting a modified malachite green colorimetric assay.^[^ ^31^ ^]^ As shown in Figure 3H v‐ATPase activity in lysosomal fractions was much lower in Lamtor5‐KO macrophages than in control cells, implying that Lamtor5‐mediated regulation of lysosomal acidification was likely through modulation of v‐ATPase activity.

Lysosomes are generally thought of as the terminal degradative machinery responsible for the elimination of macromolecules and cellular debris through the autophagic pathway. Given the lysosome‐regulating function of Lamtor5 depicted above, we then assessed its potential role in regulating the autophagic pathway. Notably, the data showed that autophagosomes, which were characterized by containing double layer membrane and electron‐dense undigested materials, but not LC3‐associated phagosomes (LAPosomes, a single layer membrane vesicle containing engulfed cargo) were preferentially accumulated in Lamtor5 KO macrophages relative to control cells (Figure 3I). In congruence, we found that the amounts of autophagosomes induced by rapamycin were increased in Lamtor5 KO macrophages, while the numbers of Zymosan‐containing LAPosome that were coated by LC3^[^ ^32^ ^]^ were not significantly changed upon Lamtor5 deletion (Figure S3, Supporting Information). The data thus indicated that Lamtor5 ablation affected the formation of canonical autophagy but not LAP. In support of this, we observed that the ratio of LC3‐II/I, a well‐defined indicator for autophagosome formation and maturation, was reduced in Lamtor5 KO macrophages even under serum starvation (Figure 3J). The protein level of p62, an autophagy substrate, as well as the amount of p62 puncta, were elevated in Lamtor5 KO macrophages (Figure 3J,K), supporting impaired autophagic pathway upon Lamtor5 loss. To directly visualize the autophagic flux, we additionally exploited a reporting system consisting of a tandem mCherry‐EGFP‐LC3B, which was designed to detect non‐acidified and acidified autophagic structures as yellow puncta and red puncta, respectively because GFP was more sensitive to acidic conditions. Strikingly, the result showed that Lamtor5 KO macrophages maintained yellow upon serum starvation, while WT cells exhibited red, indicating that starving‐induced autophagy was disrupted in Lamtor5 deficient macrophages (Figure 3L). As a control for the blocked autophagic flux, treatment of v‐ATPase inhibitor, Bafilomycin A1 (BafA1), elicited a yellow dye regardless of the presence or absence of Lamtor5 (Figure 3L). Together, the data indicated that Lamtor5 played a pivotal role in maintaining lysosomal integrity, v‐ATPase activity, and autophagic flux.

### 2.4 Lamtor5 Promotes the v‐ATPase Assembly via Interacting with ATP6V1A

**Figure 4:**
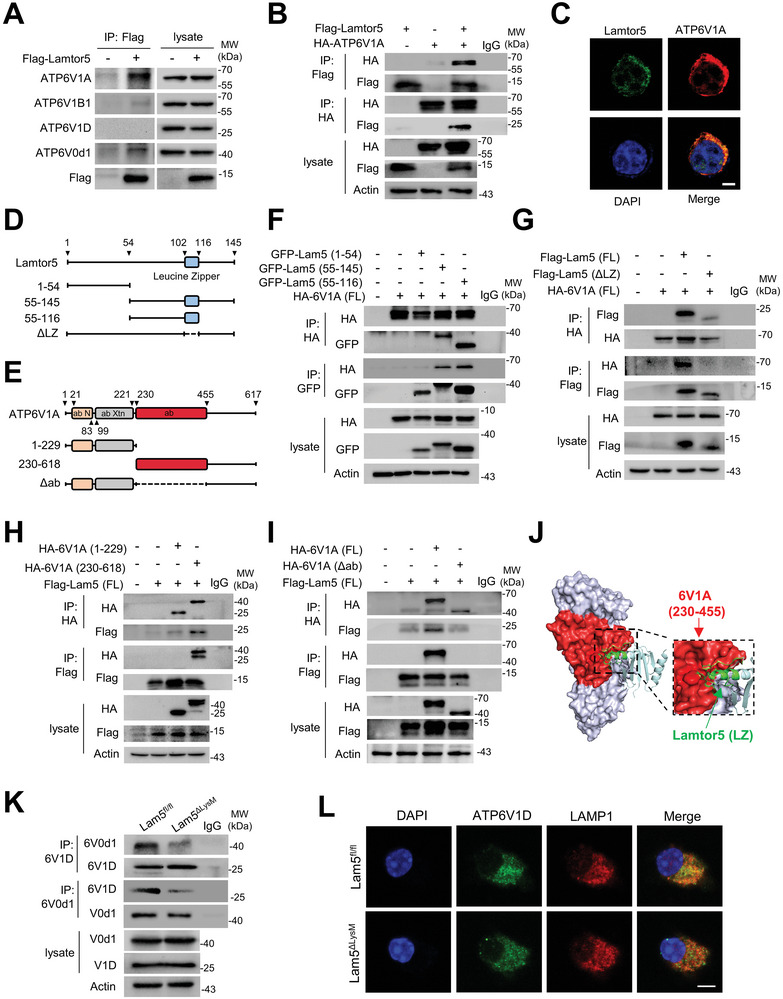
Lamtor5 promotes v‐ATPase assembly via associating with ATP6V1A. A) Co‐IP of v‐ATPase subunits by Lamtor5. 293T cells were transfected with FLAG‐Lamtor5 plasmid, and subjected to immunoprecipitation by anti‐Lamtor5 beads and immunoblotting with the indicated antibodies. IgG served as isotype control. B) Co‐IP of Lamtor5 and ATP6V1A in 293T cells that were transfected with FLAG‐Lamtor5 and/or HA‐ATP6V1A plasmids. C) Colocalization of Lamtor5 with ATP6V1A. Scale bar, 5 µm. D) Schematic illustration of Lamtor5 truncates. Δ*LZ*, the full Lamtor5 gene fragment with deletion of the leucine zipper (LZ) domain. E) Schematic illustration of ATP6V1A truncates. Δ*ab*, the full ATP6V1A gene fragment with deletion of the *ab* domain. F) Co‐IP of HA‐ATP6V1A and GFP‐tagged truncated Lamtor5. G) Co‐IP of HA‐ATP6V1A and Flag‐tagged intact or LZ‐depleting Lamtor5. H) Co‐IP of Flag‐Lamtor5 and HA‐tagged truncated ATP6V1A. I) Co‐IP of Flag‐Lamtor5 and HA‐tagged intact or ab‐depleting ATP6V1A. J) Stereoview of the ATP6V1A and Lamtor5 interaction using Z‐DOCK program. Shown is the potential interactive interface between ATP6V1A (red, *ab* domain) and Lamtor5 (green, LZ domain). K) Co‐IP of ATP6V1D with ATP6V0d1 or vice versa in macrophages from Lamtor5^ΔLysM^ and Lamtor5^fl/fl^ mice. L) Colocalization of ATP6V1D and LAMP1 in macrophages from Lamtor5^ΔLysM^ and Lamtor5^fl/fl^ mice. Scale bar, 5 µm.

Next, we sought to address how Lamtor5 modulated the activity of v‐ATPase, an ATP‐driven proton pumping that controls lysosomal pH and acidification. v‐ATPase is a multi‐subunit complex composed of cytosolic V1 sector and lysosomal membrane‐anchored V0 sector. Initially, we tested and confirmed that Lamtor5 exerted marginal effects on the level of the V1 or V0 subunits, such as ATP6V1A, ATP6V1B1, ATP6V1D, and ATP6V0d1 (Figure S4A, Supporting Information). We then explored whether Lamtor5 interacted with the v‐ATPase components and affected its assembly or stability. For this, co‐immunoprecipitation (co‐IP) was performed to identify the potentially interacting molecule(s) in HEK293T cells that were transfected with Flag‐tagged Lamtor5‐expressing plasmids. Strikingly, we found that Lamtor5 strongly co‐immunoprecipitated with ATP6V1A and much weakly with ATP6V0d1 respectively (**Figure** **4**A). The binding of Lamtor5 to ATP6V1A and vice versa was also confirmed in 293T cells exogenously expressing these two molecules (Figure 4B). Moreover, colocalization of Lamtor5 and ATP6V1A was confirmed following the immunofluorescence staining (Figure 4C).

To give more information on this interaction, we next produced a set of constructs encoding full‐length and truncated fragments of Lamtor5 or ATP6V1A respectively (Figure 4D,E). The Co‐IP assay revealed that the leucine zipper (LZ) domain of Lamtor5 (102‐116 aa) was indispensable for the association of Lamtor5 with ATP6V1A (Figure 4F,G). Also, we identified that the *ab* domain (230‐455 aa) of ATP6V1A, the structure responsible for ATPase activity,^[^ ^33^ ^]^ was essential for its ligation with Lamtor5 (Figure 4H,I). In support of this, the Z‐DOCK‐based prediction program^[^ ^34^ ^]^ identified the interface between the *ab* domain of ATP6V1A and the LZ helix of Lamtor5 as the optimal binding site (Figure 4J).

The assembly of v‐ATPase is a reversible process requiring the V1 domain to be tethered onto lysosomal membranes for assembling with the V0 domain.^[^ ^9^, ^35^ ^]^ Considering the lysosome‐tethering property of Lamtor5, we hypothesized that the binding of ATP6V1A to Lamtor5 at the lysosome might be a prerequisite for the V0/V1 assembly. Indeed, our data showed that Lamtor5 ablation substantially affected the binding of the V1 section (exemplified by ATP6V1D) to the membrane‐integral V0 domain (exemplified by ATP6V0d1) (Figure 4K), which in turn precluded the recruitment of V1 subunit (ATP6V1A or ATP6V1D) to the LAMP1^+^ lysosome (Figure 4L; Figure S4B, Supporting Information). Thus, it appeared that Lamtor5 served as a tethering factor at lysosomes to facilitate the assembly of the V0/V1 holoenzyme and hence the activity of v‐ATPase. In support, the data showed that Lamtor5 ablation caused lysosomal de‐acidification, which was corrected upon the induction of the intact but not LZ‐lacking Lamtor5‐expressing plasmids, further corroborating the requirement of the ATP6V1A/Lamtor5 interaction for v‐ATPase activity (Figure S4C, Supporting Information). We also confirmed that the v‐ATPase‐regulatory effect of Lamtor5 was essentially through ATP6V1A, as the knockdown of ATP6V1A failed to resume lysosomal acidity despite the recovery of Lamtor5 in these cells (Figure S4D, Supporting Information). Together, our data indicated that Lamtor5, by specifically associating with ATP6V1A, promoted the recruitment of the V1 to V0 subunit for v‐ATPase assembly, and thereby enabled lysosome acidification and catabolic function.

### 2.5 Lamtor5 Binding to v‐ATPase Restrains mTORC1 Activity via Interfering the Rag/mTOC1 Interaction

**Figure 5:**
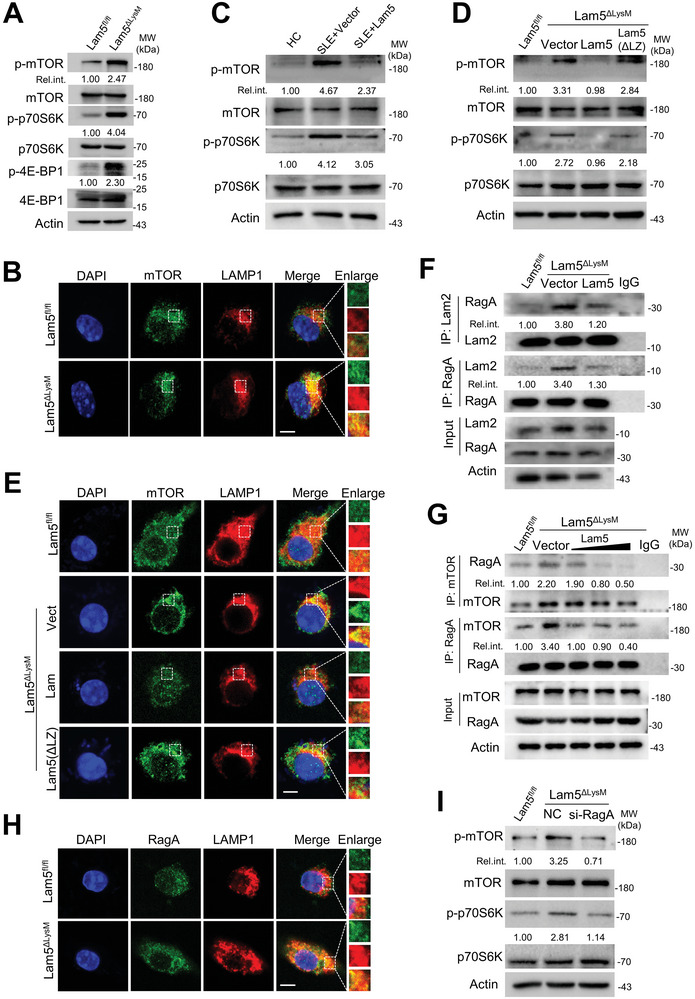
Lamtor5 binding to v‐ATPase restrains mTORC1 activation. BMDMs were prepared from 4‐month‐old Lamtor5^ΔLysM^ and Lamtor5^fl/fl^ mice for subsequent functional analysis. A) Immunoblotting for phosphorylated and total mTOR and downstream signaling molecules in macrophages. B) Immunofluorescence shows the colocalization of mTOR and LAMP1 (a marker of lysosomes). Scale bar, 5 µm. C) PBMCs of SLE patients were transfected with or without Lamtor5‐expressing plasmids and subjected to subsequent functional analysis. Immunoblotting of phosphorylated and total mTOR and p70S6K. D) The activation of mTORC1 pathway, and E) lysosomal localization of mTOR in Lamtor5 sufficient or Lamtor5 deficient macrophages that were transfected with the intact or LZ‐lacking Lamtor5‐expressng plasmids respectively. Scale bar, 5 µm. F) The effect of Lamtor5 on the association of Lamtor2 and RagA. The interaction of Lamtor2 and RagA was detected by co‐immunoprecipitation in Lamtor5^fl/fl^ and Lamtor5^ΔLysM^ macrophages transfected with control or Lamtor5‐expressing plasmids respectively. IgG was used as a control. G) The effect of Lamtor5 on the association between mTOR and RagA in macrophages. BMDMs from 4‐month‐old Lamtor5^Δ^ *^LysM^* were transfected with empty vectors or Lamtor5‐expressing plasmids at the indicated dosage. The association of RagA and mTOR was determined by co‐IP. BMDMs from Lamtor5^fl/fl^ mice and IgG were used as the controls. H) Lysosomal residency of RagA in Lamtor5 sufficient of deficient macrophages was determined by co‐localization of RagA and LAMP1. Scale bar, 5 µm. Shown are representative images from two or three independent experiments. I) The activation of mTORC1 pathway in Lamtor5 sufficient macrophages, or Lamtor5 KO macrophages that were transfected with RagA siRNA or negative control (NC). The relative band densities normalized to their corresponding protein input were shown.

Lysosomes are not only a cellular catabolic system but also a molecular platform for the residency and activation of multiple signaling molecules involving the mTORC1 kinase. Intriguingly, our study demonstrated that Lamtor5 ablation caused enhanced phosphorylation of mTORC1, as well as the subsequent S6 kinase (S6K) and eukaryotic translation initiation factor 4E‐binding protein (4E‐BP) in macrophages under the steady state (**Figure** **5**A). In parallel, translocation of mTORC1 to the lysosome, a critical step for its activation, was markedly increased upon Lamtor5 loss (Figure 5B). Combined with the finding about the blunted autophagic pathway in Lamtor5^−/−^ macrophages (Figure 3I**–**L), the process causatively related to mTORC1 activation, we proposed that Lamtor5 exerted a negative role in regulating mTORC1 activation. Supportively, we also found that PBMCs from SLE patients exhibited enhanced activation of mTORC1 compared with that from healthy controls, and importantly, enforced expression of Lamtor5 remarkably decreased this undesirable mTORC1 activation (Figure 5C). We then sought to address how Lamtor5 modulated mTORC1 activity. Notably, the data showed that the inhibition of Lamtor5 on mTORC1 phosphorylation and lysosomal translocation was largely abrogated when its LZ domain was deleted (Figure 5D,E). Since the LZ domain was necessary for Lamtor5 binding to v‐ATPase, it was likely that the association of Lamtor5 with v‐ATPase was essential for restraining mTORC1 in normal conditions. It is currently known that mTORC1 activation requires the Ragulator complex as a scaffolding factor for Rag GTPase tethering, which in turn recruits mTORC1 onto lysosomes for activation. As Lamtor5 was not able to associate directly with mTORC1 revealed by us and other investigators,^[^ ^36^, ^37^ ^]^ a hypothesis for this mTORC1 regulation might be that Lamtor5 binding to v‐ATPase interfered with the Ragulator/Rag association and/or the Rag/mTOR1 interaction. Critically, our data showed that disruption of the Lamtor5/ATP6V1A interaction by Lamtor5 deletion caused enhanced interaction of Ragulator (Lamtor2) and Rag GTPase (RagA), while overexpression of Lamtor5 weakened this binding, indicating that the association of Lamtor5 with v‐ATPase precluded the Ragulator and Rag GTPase ligation, an essential step for mTORC1 activation (Figure 5F). Interestingly, no significant alteration was observed in the association of Lamtor1 with RagA (Figure S5, Supporting Information), supporting the specific effect of Lamtor5. This reason was likely due to Lamtor2 (not Lamtor1) being situated between Lamtor5 and Rag GTPase.^[^ ^36^, ^38^ ^]^ On the other hand, we showed that Lamtor5 deletion strengthened the RagA and mTOR association, and restoration of Lamtor5 resulted in a weakened binding of RagA with mTOR in Lamtor5 KO macrophages (Figure 5G). Accordingly, the loss of Lamtor5 enhanced lysosomal tethering of RagA and hence mTORC1 activation (Figure 5H,I). This enhanced mTORC1 activity was compromised upon RagA deletion (Figure 5I), further supporting the involvement of the Rag GTPase in the regulation. Together, our data indicated that Lamtor5, via ligation of v‐ATPase, precluded the interaction between Ragulator, Rag GTPase, and mTORC1, and thereby impeded lysosomal tethering of Rag GTPase to prevent undesirable mTORC1 activation.

### 2.6 Re‐Acidification of Lysosomes or Inhibition of mTORC1 Ameliorates the Autoimmune Phenotype Associated with Lamtor5 Defect

**Figure 6:**
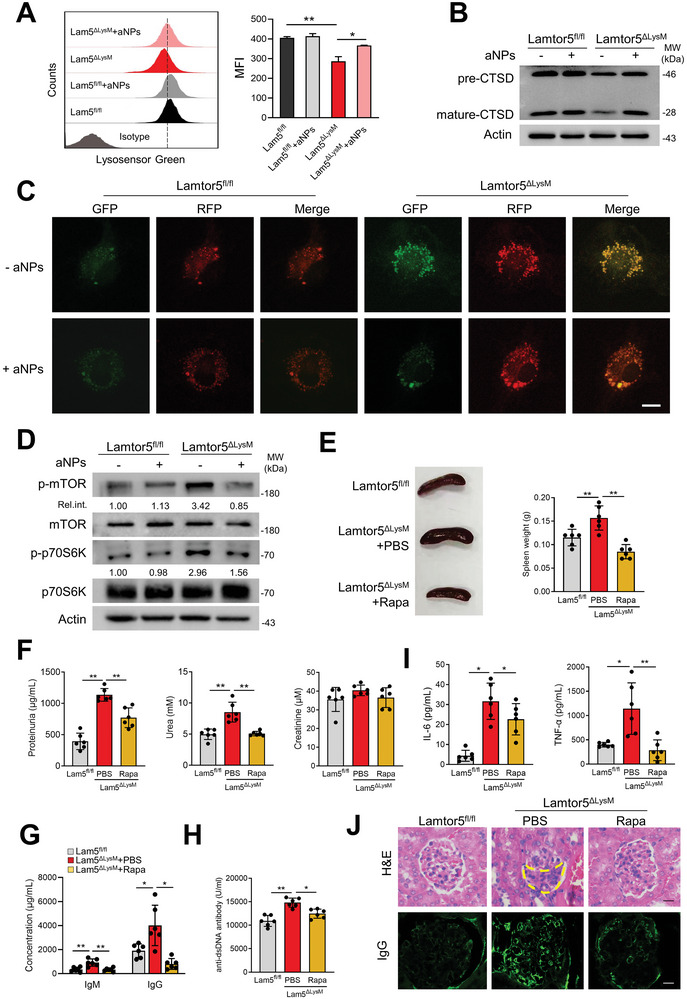
Re‐acidification of lysosomes or inhibition of mTORC1 attenuates autoimmune pathology associated with Lamtor5 loss. A–D) BMDMs from 4‐month‐old Lamtor5^ΔLysM^ and Lamtor5^fl/fl^ mice were treated with or without aNPs (3 mg mL^−1^) for 4 h, and subjected to functional analysis. FACS analysis of macrophages stained with LysoSensor Green DND‐189. Relative fluorescence quantification was shown; Immunoblotting for precursor or mature CTSD in macrophages; Immunofluorescence staining of macrophages transfected with mCherry‐EGFP‐LC3B plasmids, followed by FBS starvation for 24 h. Scale bar, 5 µm; Immunoblotting for phosphorylated and total mTOR and p70S6K. The relative intensity of targeted protein bands normalized to their corresponding input was provided. E–I) Rapamycin (2 mg kg^−1^) or PBS was administrated to Lamtor5^ΔLysM^ mice (4‐month‐old, n = 6) by intraperitoneal injection every day for 2 weeks. Lamtor5^fl/fl^ mice (4‐month‐old, n = 6) were used as control. Representative images and weight of spleens; The concentrations of proteinuria, and serum urea and creatinine; Serum levels of IgM and IgG and anti‐dsDNA autoantibody; Serum levels of IL‐6 and TNF‐α (I). J) Rapamycin (2 mg kg^−1^, i.p.) or PBS was administrated to Lamtor5^ΔLysM^ mice (8‐month‐old, n = 6) every day for 2 weeks. Lamtor5^fl/fl^ mice (8‐month‐old, n = 6) were used as control. H&E staining or immunofluorescence staining of kidney sections (Alexa Fluor 488‐conjugated anti‐mouse IgG) was shown. The dotted area represents the crescentic lesion. Scale bar, 20 µm. The data are from three independent experiments. All results are expressed as the mean ± SD. ^*^ *P* < 0.05, ^**^ *P* < 0.01 by Student's *t* test.

The above data indicated that myeloid Lamtor5 ablation caused lysosomal de‐acidification and unwanted mTORC1 activation, leading to blunted autophagic flux and defective lysosomal degradation, and hence spontaneous inflammation and autoimmunity in Lamtor5^ΔLysM^ mice. We therefore wondered whether the restoration of defective lysosomal pH in macrophages would correct the associated immunopathology. To test it, we constructed PolyDL‐lactic‐co‐glycolic acid (PLGA)‐based acidic nanoparticles (aNPs), an FDA‐approved agent designed to target lysosomes and restore lysosomal pH.^[^ ^39^, ^40^ ^]^ The particle size analyzer revealed that PLGA‐aNPs adopted a spherical shape with a diameter of ≈180 nm (Figure S6A, Supporting Information). Labeled by Nile red, aNPs were shown to be internalized into murine macrophages and localized at LAMP1^+^ lysosomes (Figure S6B, Supporting Information). We therefore proceeded to test the consequence of re‐acidification of lysosome in Lamtor5 deficient macrophages. The result showed that Lamtor5 KO macrophages, by taking PLGA‐aNPs, corrected the impaired lysosomal pH and CTSD maturation to a level comparable to Lamtor5^+/+^ cells, (**Figure** **6**A,B). Moreover, the compromised autophagic flux in Lamtor5 lacking cells was largely restored upon aNPs treatment, as demonstrated by mCherry‐EGFP‐LC3B staining (Figure 6C). Along with this, enhanced mTORC1 activation and production of pro‐inflammatory factors induced by Lamtor5 loss were abated upon re‐acidification of lysosomes (Figure 6D; Figure S6C, Supporting Information). The results thus indicated that restoration of defective lysosomal acidification by PLGA‐aNPs treatment substantially dampened the inadvertent mTORC1 activation and inflammatory responses caused by Lamtor5 ablation.

Given the central importance of deregulated mTORC1 in inflammatory and autoimmune diseases, we therefore assessed whether inhibition of mTROC1 pathway would rectify the immunopathology associated with Lamtor5 loss in mice. For this, rapamycin, a well‐characterized mTORC1 inhibitor and also an approved prescription for patients with active SLE, was applied in the study. Strikingly, the results demonstrated that rapamycin treatment mitigated splenomegaly (Figure 6E), reduced serum levels of urine protein and proteinuria, and decreased the concentration of IgM/G and anti‐dsDNA autoantibodies in Lamtor5^ΔLysM^ mice (Figure 6F–H). Also, the generation of proinflammatory cytokines such as IL‐6 and TNFα was repressed upon rapamycin administration (Figure 6I). Moreover, treatment of mTOC1 inhibitor substantially alleviated the glomerulonephritis‐like pathology in the kidneys of Lamtor5^ΔLysM^ mice (Figure 6J). Together, the data proved that re‐acidification of lysosomes and inhibition of the mTORC1 pathway rectified the immunopathology associated with the Lamtor5 defect, reinforcing the central role of Lamtor5 in coordinating these two major machineries.

## 3 Discussion

In the present study, we identify Lamtor5 as a critical factor for immune homeostasis and preventing autoimmune diseases. The pathogenic role of defective Lamtor5 is confirmed by not only the immunopathology in myeloid Lamtor5 KO mice but also the clinical manifestation in SLE patients. We provide the first evidence to show that Lamtor5 is specifically associated with ATP6V1A subunit to promote the assembly of the V0/V1 holoenzyme, enable v‐ATPase activity and lysosomal activation, and thereby facilitate the autophagic flux. On the other hand, binding of Lamtor5 to v‐ATPase affected the association of Ragulator with Rag GTPase and subsequently precluded lysosomal tethering of Rag GTPase to recruit mTORC1 for activation. We thus established Lamtor5 as an orchestrator that links v‐ATPase, auto‐lysosome, and mTORC1 pathways to self‐control the immune reaction and avoid undesirable responses. The findings elucidate an unappreciated mechanism that controls immune homeostasis and unveil a potential therapeutic target for SLE and probably other autoimmune diseases.

Lamtor5 is one of the subunits of the Ragulator complex, which is a pentameric protein structure serving as a scaffold to mediate mTORC1 activation via Rag GTPase. Despite their canonical roles, recent studies have demonstrated that the Ragulator components, such as Lamtor1 and Lamtor2, have distinct and vital functions.^[^ ^20^ ^]^ We herein demonstrate that Lamtor5 played an indispensable role in controlling macrophage activation and immune homeostasis, with loss of Lamtor5 leading to aberrant immune activation, autoinflammation, and SLE‐like manifestation. The systemic immunopathology is causatively related to aberrant activation of proinflammatory monocytes/macrophages both in Lamtor5 KO mice and SLE patients. Emerging evidences have demonstrated that dysfunctional lysosomes and associated with accumulation of immune complex (IC) and cellular debris would cause phagosomal membrane permeabilization, leading to dsDNA and autoantibodies leakage into the cytosol for activating the immune sensors such as NLRP3 and STING, which would subsequently promote proinflammatory macrophages polarization and SLE pathogenesis.^[^ ^41^, ^42^ ^]^ In line with this notion, our data identify a lysosome‐centered and macrophage‐targeted mechanism as a contributing factor for the observed immunopathology associated with Lamtor5 ablation. Although at this stage, we cannot completely exclude the involvement of other myeloid cells, the experiments exploiting macrophage depletion supported that dysfunctional macrophages induced by Lamtor5 loss play a dominant role in this autoimmune pathology. Moreover, we observed that the level of macrophage Lamtor5 was significantly decreased in patients with SLE compared with healthy subjects, and this reduction is conversely related to the renal pathological grading (Figure 1I). Thus, our data consistently bolster the importance of macrophages in Lamtor5‐associated pathophysiology, adding Lamtor5 to the expanding spectrum of autoimmune‐related factors.^[^ ^43^, ^44^, ^45^ ^]^

One novel discovery of our current study is to identify ATP6V1A as a genuine target for Lamtor5 to modulate lysosome function. As is known, the assembly of the V0/V1 holoenzyme of v‐ATPase is a reversible process, with the V0 sector embedded in lysosomal membranes and the V1 domain switching between the lysosomal membrane and the cytosol depending on intracellular conditions.^[^ ^46^ ^]^ Currently, little is known about how the V1 subunits are recruited and anchored at the lysosomal membrane to form v‐ATPase. Our study reveals that Lamtor5 promoted the V0/V1 assembly via specific association with ATP6V1A, as deletion of Lamtor5 or disruption of the ATP6V1A/Lamtor5 interaction substantially impeded lysosomal tethering of V1 and weakened the V0/V1 assembly. Due to the central importance of lysosomes in governing vital activities and the reversibility of the v‐ATPase assembly, new regulators targeting v‐ATPase have recently been studied.^[^ ^14^, ^47^, ^48^ ^]^ Accordingly, small molecules potentially binding to v‐ATPase and regulating lysosomal pH are currently discovered to treat relevant diseases.^[^ ^49^, ^50^ ^]^ Similar to our current study, EN6 was newly discovered to covalently interact with ATP6V1A to promote v‐ATPase activity and lysosome acidification, and simultaneously inhibit mTORC1 activity (50). The data imply that ATP6V1A might be an accessible target for modulating v‐ATPase activity and lysosomal function.

Somewhat unexpectedly, our data demonstrate that enhanced mTORC1 activity was induced by Lamtor5 ablation in macrophages, which seemingly contradicting with the previous report that the Lamtors‐containing Ragulator was required for mTORC1 activation in response to the amino acid.^[^ ^51^ ^]^ We sought to address the paradox by examining the effect of Lamtor5 ablation on the Rag/mTORC1 association and the Ragulator/Rag association, the two major events driving the activation of the mTOR pathway. Remarkably, our data showed that loss of Lamtor5 significantly boosted the interaction of Ragulator (Lamtor2) and Rag GTPase (RagA), and resumption of Lamtor5 in Latmor5 KO macrophages largely abrogated this enhancement. That data indicates that Lamtor5 substantially affected the Ragulator/Rag interaction. Since previous studies have shown that Lamtor2 is a specific intermediator linking Lamtor5 and RagA,^[^ ^36^, ^38^ ^]^ it is rational that deletion of Lamtor5 affects the binding of Lamtor2, not Lamtor1 to Rag GTPase, as revealed by our study (Figure 5F; Figure S5, Supporting Information). This may suggest a competitive binding model wherein Lamtor5 competes with RagA to associate with Lamtor2, leading to either Lamtor5/RagA binding to enable v‐ATP assembly and lysosome integrity, or Ragulator/Rag association to promote mTORC1 activation. However, additional experiments, such as the binding dynamics and binding affinity measuring, are needed to confirm this notion. On the other hand, as the loss of Lamtor5 significantly strengthened the Ragulator/Rag association, it is expected that this enhanced binding would cause more Rag GTPase tethering at the lysosome with intensified binding force, which in turn attracts more mTORC1 for enhanced activation. This provides a plausible explanation for the boosted effects of Lamtor5 loss on mTORC1 activation and suggests an unappreciated mechanism that coordinates the interaction among Ragulator, Rag GTPase, and mTORC1 to dynamically regulate catabolic (lysosome) and anabolic (mTOR) pathways.

From another aspect, we would like to mention that the previous report about the requirement of Ragulator for mTOR activation is based on the instant cellular response to amino acids,^[^ ^20^ ^]^ whereas our system is focused on the homeostatic function of Lamtor5 with no nutrimental interference. In fact, we previously reported that the addition of leucine, an essential branch‐chain amino acid known to activate the mTOR pathway, suppressed the expression of Lamtor5, whereas deprivation of leucine or serum elevated Lamtor5 level.^[^ ^24^ ^]^ This suggests that nutritional factors may act through Lamtor5 to regulate mTOR activity and subsequent macrophage status. The observations, although preliminary, potentially support the recommendation of low‐protein diets for patients with SLE or other diseases.^[^ ^52^, ^53^ ^]^ This topic merited further investigation to better understand the metabolic regulation of inflammatory responses and may provide conductive clues for SLE treatment.

In conclusion, our study establishes Lamtor5 as a critical regulator for maintaining immune homeostasis and preventing autoimmunity via coordinating v‐ATPase, lysosome, and mTORC1 activity, which might open up a new avenue for treating autoimmune disorders.

## 4 Experimental Section

The experimental section was included in the supplementary materials.

## Conflict of Interest

The authors declare no conflict of interest.

## Author Contributions

W.Z. and Z.S. contributed equally to this work. L.S. designed the research project. W.Z., Z.S., Y.T., C.J., W.G., N.L., and L. Y. performed experiments and analyzed data. C.C., S.L., F.X., and D.W. contribute to experimental material and insightful suggestions. L.S. and W.Z. wrote the manuscript.

## Supporting information

Supporting Information

## Data Availability

The data that support the findings of this study are available from the corresponding author upon reasonable request.
