# Supplementary material for: Defective Lamtor5 Leads to Autoimmunity by Deregulating v‐ATPase and Lysosomal Acidification
Source: Adv Sci (Weinh). 2024 Apr 19;11(22):2400446. doi: 10.1002/advs.202400446 (PMC11165510; doi:10.1002/advs.202400446)
Supplement: Supplementary file 1 — Supporting Information [file ADVS-11-2400446-s001.pdf]

ADVANCED SCIENCE
Open Access

Supporting Information

for *Adv. Sci.*, DOI 10.1002/advs.202400446

Defective Lamtor5 Leads to Autoimmunity by Deregulating v-ATPase and Lysosomal Acidification

Wei Zhang, Zhou Sha, Yunzhe Tang, Cuiyuan Jin, Wenhua Gao, Changmai Chen, Lang Yu, Nianyin Lv, Shijia Liu, Feng Xu, Dandan Wang* and Liyun Shi**

Supporting Information

Defective Lamtor5 leads to autoimmunity by deregulating v-ATPase and lysosomal acidification

Wei Zhang, Zhou Sha, Yunzhe Tang, Cuiyuan Jin, Wenhua Gao, Changmai Chen, Lang Yu, Nianyin Lv, Shijia Liu, Feng Xu*, Dandan Wang*, Liyun Shi*

Experimental Section

Materials

Clinical samples

Systemic Lupus Erythematosus (SLE) disease subjects and healthy donors were recruited from the Department of Rheumatology at Nanjing Drum Tower Hospital. All studies were reviewed and approved by the Ethics Committee at The Affiliated Drum Tower Hospital of Nanjing University Medical School (2009004 and 2016-027-01). All SLE disease subjects and healthy donors were recruited with informed consents. Blood samples were diluted 1:2 in cold PBS and PBMCs isolated by density gradient centrifugation using Peripheral blood mononuclear Cell Isolation Kit (P8680, Solarbio).

Reagents and antibodies

Bafilomycin A1 (S1413) was obtained from Selleck Chemicals. Dynabeads™ Protein A for Immunoprecipitation (10002D), LysoSensor Green DND-189 (L7535), BODIPY FL

Pepstatin A (P12271), Intracellular pH Calibration Buffer Kit (P35379) and DQ-BSA (D-12050) were purchased from Thermo Fisher Scientific. Mouse dsDNA-Ab ELISA Kit (ml063514) was purchased from mlbio. Mouse IgM ELISA Kit (70-EK276-96) and Mouse IgG ELISA Kit (70-EK271-96) were purchased from MULTI SCIENCES. Rheumatoid Factor (KH1053-2) and β2-microglobulin (KH1023-4) Test Kits were purchased from KINGSBIO. LysoTracker DND-99 (C1046) was obtained from Beyotime and LysoSensor Yellow/Blue DND-160 (40767ES50) was from YEASEN. Cell/Tissue lysosome Isolation Kit (GMS10118.3) was obtained from GENMED SCIENTIFICS and Malachite Green Phosphate Assay Kit (POMG-25H) was from BioAssay System.

Abs against Lamtor5 (ab157480), LAMP1 (ab25245, ab208943), Cathepsin D (ab75852), ATP6V1A (ab199326), ATP6V1B1 (ab192612), ATP6V1D (ab157458), ATP6V0D1/P39 (ab202899) and p62 (ab56416) were from Abcam. Abs against GFP (#2956 and #2955), HA-Tag (#3724), LC3A/B (#4108), LC3B (#3868), LAMP1 (#3243), SQSTM1/p62 (#39749), β-actin (#3700), p-mTOR (Ser2448, #5536), mTOR (#2972), p-ULK1(Ser317, #12753), ULK1 (#8054), p-p70S6 Kinase (Thr421/Ser424, #9204), p70S6 Kinase (#2708), p-4E-BP1(Thr37/46, #2855) and 4E-BP1 (#9644) were obtained from Cell Signaling Technology. The Anti-DDDDK-tag (M185-3L) was purchased from MBL. The HRP-conjugated secondary antibody was purchased from Thermo Fisher Scientific. Alexa Fluor-conjugated antibodies were from eBioscience.

Expression plasmids encoding Lamtor5 (pCMV-tag2B-Lamtor5), ATP6V1A (pCDNA3.1-ATP6V1A) and truncated ATP6V1A were constructed by KeyGEN BioTECH. The tandem mCherry-EGFP-LC3B plasmid was bought from addgene. Expression plasmids

encoding truncated Lamtor5 proteins were constructed by GeneChem. Ctrl shRNA and sh-Lamtor5 plasmids were kind gifts from Dr. Hang Zhang (Hangzhou Normal University).

Mice

Lamtor5$^{fl/fl}$ mice were constructed by CaseGene Biotech company and crossed with LysM-Cre transgenic mice (The Jackson Laboratory; JAX no. 004781). Their progeny (Lamtor5$^{fl/+}$LysM-Cre) were bred to obtain Lamtor5$^{fl/fl}$LysM-Cre mice. All mice were maintained under a 12-hour light-dark cycle with *ad libitum* access to regular food and water. All animal experiments were approved by the Ethical Review Committee of NJUCM (20200326).

Methods

Cell culture

HEK293T cells and RAW264.7 cells were maintained in Dulbecco's Modified Eagle Medium (DMEM) supplemented with 10% fetal bovine serum, 100 U/mL penicillin and 100 mg/mL streptomycin at 37℃ in 5% CO_2. Mouse bone marrow macrophages were extracted from the femurs of both hind legs and cultured in DMEM containing 10% fetal bovine serum and M-CSFs (315-02, Peprotech) at 25 ng/mL.

Macrophage depletion

Female Lamtor5$^{fl/fl}$ mice and Lamtor5$^{\Delta LysM}$ mice (8 months old, n=5) were treated with either vehicle or clodronate liposomes (CLs, 100 μg/mouse) twice a week for 1 month. Blood, urine, and kidney samples were collected for subsequent analysis.

Cytokine measurement

Serum samples from mice were harvested and Luminex-based multiplexed fluorescence assay was used to assess cytokines and chemokines using the Bio-Plex Pro Mouse Cytokine Group I Panel 23-Plex Assay kit (Bio-Rad).

Analysis of functional markers

Serum levels of rheumatoid factor were quantified by clinical biochemical test kits (KINGSBIO) using a AU480 Chemistry Analyzer (Beckman Coulter).

ELISA

Serum dsDNA-Ab, IgG and IgM were measured according to manufacturer's instructions, by commercially available ELISA assays. Briefly, 10 µL of diluted sera were incubated for 30 minutes on the ELISA plates, and after three washing steps, the horseradish peroxidase labeled antibodies were added as a conjugate and incubated at room temperature for 15 minutes. After three additional washing steps, 100 µL of tetramethylbenzidine (TMB) were added and the samples were further incubated for 15 minutes before stopping the reaction with 100 µL 1M H_2SO_4. Optical density (OD) was measured at 450 nm. All assay procedures were performed automatically on the Scientific Microplate Reader (PerkinElmer, USA).

Autophagic flux analysis

BMDMs were transfected with tandem mCherry-EGFP-LC3B plasmid for 48 h. Cells were treated as indicated. The cells on coverslips were washed twice with PBS, fixed with 4% paraformaldehyde for 15 min. After mounting, fluorescence images were acquired using a confocal laser-scanning microscope.

Flow cytometry

To evaluate the functional lysosomes, the probe of LysoSensor Green DND-189, which fluoresces in an acidic environment (pH ≤ 5.2), was used. BMDMs were loaded with 1 μM of LysoSensor in pre-warmed medium for 30 min at 37 °C, and then washed twice with PBS and immediately analyzed by flow cytometry. The data were analyzed with FlowJo (Tree Star). For DQ-BSA quench assay, BMDMs were treated with 100 μg/ml of DQ Green BSA (molecular probe) at 37 °C for 2 h. The samples were resuspended in the staining buffer and analyzed by FACSCalibur.

Induction and detection of LC3-Associated Phagocytosis (LAP)

BMDMs from 4-month-old Lamtor5$^{\Delta LysM}$ and Lamtor5$^{fl/fl}$ mice were transfected with RFP-LC3 plasmid for 48h. Cells were left untreated (NS) or cultured with 200 nM rapamycin or Alexa Fluor 488-Zymosan A. RFP-LC3 puncta were assessed at 18 h post incubation, and translocation of RFP-LC3 to LAPosomes was assessed at 1 h using Laser confocal microscope.

Isolation of lysosomes and detection of v-ATPase activity

Isolation of lysosomal fraction from BMDMs was performed using differential centrifugation followed by density gradient centrifugation (Cell/Tissue lysosome Isolation Kit). The activity of v-ATPase was further determined by measuring the acid phosphatase activity with Malachite Green Phosphate Assay Kits (BioAssay Systems, POMG-25H). It is based on quantification of the green complex formed between Malachite Green, molybdate and free orthophosphate (Pi). Freshly isolated lysosomal fractions were incubated with ATP substrate and the rapid color formation was measured under a wavelength of 620 nm.

Immunofluorescence staining

Cells were grown on cover glasses, fixed with 4% paraformaldehyde, and permeabilized with 0.1% Triton X-100 in PBS. After blocked with 5% BSA, cells were stained by incubation with primary antibodies for 12 h at 4 °C. After washing, samples were incubated with Alexa Fluor-conjugated antibodies. Cell nuclei were visualized with DAPI (Sigma). Slides were mounted with SlowFade Gold anti-fade reagent (Invitrogen), and detected under the Leica TCS SP8 STED Microscope.

Transmission electron microscope (TEM)

Cells were first fixed with 2.5% glutaraldehyde in PBS (0.1M, pH7.0) for 4 h, washed, and post-fixed with 1% OsO_4 for 2 h. The specimen was dehydrated by a graded series of ethanol for 20 min at each step, and transferred to absolute acetone for 20 min. The specimen was then placed in the mixture of absolute acetone and resin overnight, and embedded in resin at 70℃ for more than 9 h. The specimen was then sectioned in Leica EM UC7 ultratome and stained respectively with uranyl acetate and alkaline lead citrate for 5-10 min. Electron micrographs were taken on JEOL Model JEM-1230 TEM.

Co-immunoprecipitation assay

For immunoprecipitation studies, cell lysates were incubated at 4°C for 2 h with a capture antibody or a control IgG, followed by overnight incubation with Dynabeads™ Protein A. The immunocomplexes were collected using the magnetic stand, washed with ice-cold PBST (PBS-0.02% Tween-20), and separated by SDS-PAGE. The samples were detected by the standard immune-blotting methods.

Structural modeling and molecular docking

The structure models of mouse Lamtor5 and mouse ATP6V1A subunit were provided by

homologous modeling web server SWISS-MODEL through submitting the amino acid sequences from NCBI respectively (Gene ID of mLamtor5: 68576; Gene ID of mATP6V1A: 11964). The interaction model between mLamtor5 and mATP6V1A was predicted by ZDOCK Server. The 3D protonation and energy minimization were done with compute option and the interaction complexes with the top ten docking scores were further evaluated. PyMOL was used to analyze and label the interaction details of structure models.

Rapamycin treatment *in vivo*

Rapamycin was dissolved in DMSO and diluted in PBS. For in vivo treatment experiments, rapamycin (2 mg/kg) or PBS was administered to Lamtor5$^{\Delta LysM}$ mice (4 months old) by intraperitoneal injection every day for 2 weeks. After 2 weeks of treatment, mice were euthanized and their sera, urine and spleen were collected for further analysis.

Lysosomal pH measurement

Quantification of lysosomal pH was performed using a ratiometric lysosomal pH dye, LysoSensor Yellow/Blue DND-160. The pH calibration curve was generated as described previously. Briefly, BMDMs from Lamtor5$^{fl/fl}$ and Lamtor5$^{\Delta LysM}$ mice were trypsinized and labeled with 1 μg/mL Lysosensor Yellow/Blue DND-160 for 30 min at 37℃ in regular medium, and excessive dye was washed out using PBS. The labeled cells were treated with 25 mM 2-(N-morpholino) ethanesulfonic acid (MES) calibration buffer (pH 4.5-7.5) containing10 mM monensin and 10 mM nigericin for 10 min and observed using a fluorescence microscopy. Light emitted at 440 and 540 nm in response to excitation at 329 and 384 nm were measured, respectively. The ratio of light emitted with 329 and 384 nm

excitation was plotted against the pH values in MES buffer, and the pH calibration curve for the fluorescence probe was generated from the plot.

Nanoparticle Tracking Analysis

PLGA nanoparticles were synthesized based on previously published protocols. Particles (10 mg) were dissolved in ddH_2O (1 ml), sonicated at low frequency for 3 minutes and filtered. NTA measurements were performed with a NanoSight NS300 (NanoSight, Amesbury, United Kingdom). Three 60-sec videos were recorded of each sample with camera level. All measurements were performed at room temperature.

RNASeq

RNA was extracted from BMDMs using Trizol extraction and Purelink RNA kit (ThermoFisher Scientific). Bioanalyzer chips were used to assess RNA quality prior to RNA sequencing. 75 bp single-end sequencing was performed on an Illumina HiSeq 2000 machine (Illumina) following the manufacturer's instructions. Differential expression was tested for normalized count data using the R package edge R, with a generalized linear model. Genes with $| \log_2 \text{(fold change)} | \geq 1$ and $P \leq 0.05$ were identified as differentially expressed genes.

Data availability

The RNA sequencing data from this publication have been deposited to the Gene Expression Omnibus database (GSE227039). The raw data supporting the conclusions of this article will be made available by the authors, without undue reservation.

Statistical analyses

All statistical calculations were performed using GraphPad Prism. Tests between two groups used two-tailed Student's *t* test. Tests between multiple groups used one-way analysis

of variance (ANOVA) with Tukey multiple comparisons. Tests between multiple groups over time used two-way ANOVA with Dunnett's multiple comparisons. Criteria for excluding samples include failure to meet quality control standards, such as insufficient sample volumes, unacceptable levels of contaminants and poor histological quality.

Table S1 Clinical characteristics of lupus patients and healthy controls

	Healthy controls	SLE patients	*p* value
Number	29	41	-
Gender (female/male)	25/4	38/3	0.429
Age (years)	47.5±7.3	mean 45.2±16.3	0.482
Disease duration (month)	NA	121.3 (1-444)	-
Baseline SLEDAI score	NA	6.6 (1-13)	-

NA, not available.

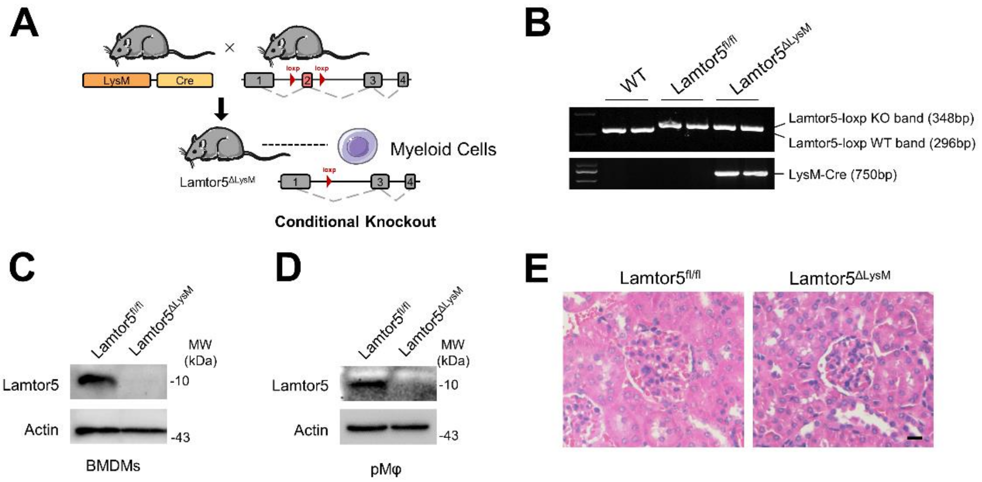

Figure S1. Generation of Lamtor5 conditional knockout mice. (A) Diagram of Lamtor5 conditional knockout mice. Numbers indicate the exons of Lamtor5. Flanking exon 2 of Lamtor5 with loxP sites generated the floxed allele (Lamtor5$^{fl/fl}$). The Lamtor5$^{fl/fl}$ mice are bred with LysM-Cre expression mice. Cre recombinase catalyzes excision of the loxP-flanked DNA sequences, resulting in a targeted deletion and leaving behind a single loxP site. **(B)**

Genotyping of Lamtor5 conditional knockout mice by PCR. **(C, D)** Knockout efficiency of Lamtor5 in BMDMs and peritoneal macrophages (pMφ) from Lamtor5$^{fl/fl}$ and Lamtor5$^{\Delta LysM}$ mice were examined by western blot. **(E)** H&E staining of kidney sections from 4-month-old Lamtor5$^{fl/fl}$ or Lamtor5$^{\Delta LysM}$ mice. Scale bar, 20 μm.

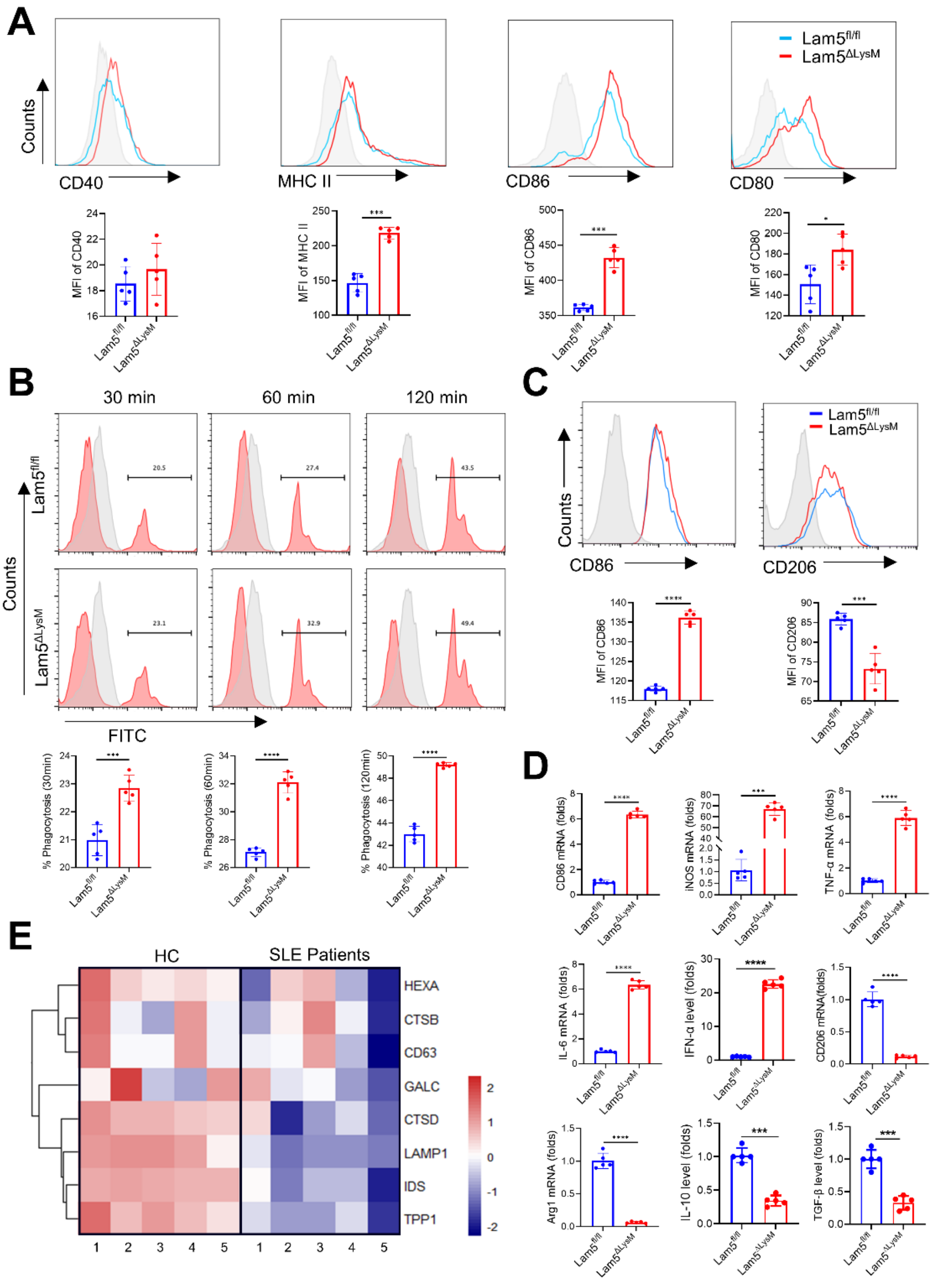

Figure S2. Loss of Lamtor5 leads to enhanced activation and pro-inflammatory phenotype of macrophages. Bone marrow cells (4 × 10^7) from 4-month-old Lamtor5$^{\Delta LysM}$ and Lamtor5$^{fl/fl}$ mice were cultured in the presence of M-CSF (25 ng/ml) for 6 d. **(A)** Flow

cytometry of surface markers of macrophages; **(B)** Phagocytic capability of macrophages. Monodisperse fluorescent microspheres were incubated with macrophages for 30, 60 and 120 min. The uptake capability of macrophages was analyzed by flow cytometry; **(C)** The expression of CD86 (M1 marker) and CD206 (M2 marker) in the BMDMs was detected by Flow cytometry. **(D)** qPCR assay of the indicated cytokines in BMDMs. Shown are representative images and the data from three independent experiments are expressed as means ± SD, with three technical replicates. **P* < 0.05, ***P* < 0.01, ****P* < 0.001, ****P* < 0.001 by student's *t* test.

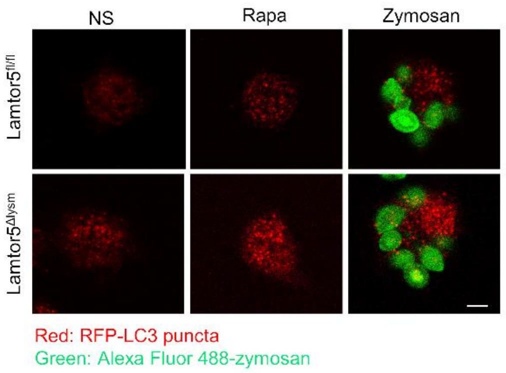

Figure S3. The effect of Lamtor5 on canonical autophagy and LAP pathway. BMDMs from 4-month-old Lamtor5$^{\Delta LysM}$ and Lamtor5$^{fl/fl}$ mice were left untreated (NS) or cultured with 200 nM rapamycin, and then Alexa Fluor 488-Zymosan A was added. RFP-LC3 puncta was assessed at 18 h post incubation, and the translocation of RFP-LC3 to LAPosomes was assessed upon Zymosan A addiministration1 h later. Scale bar, 5 μm.

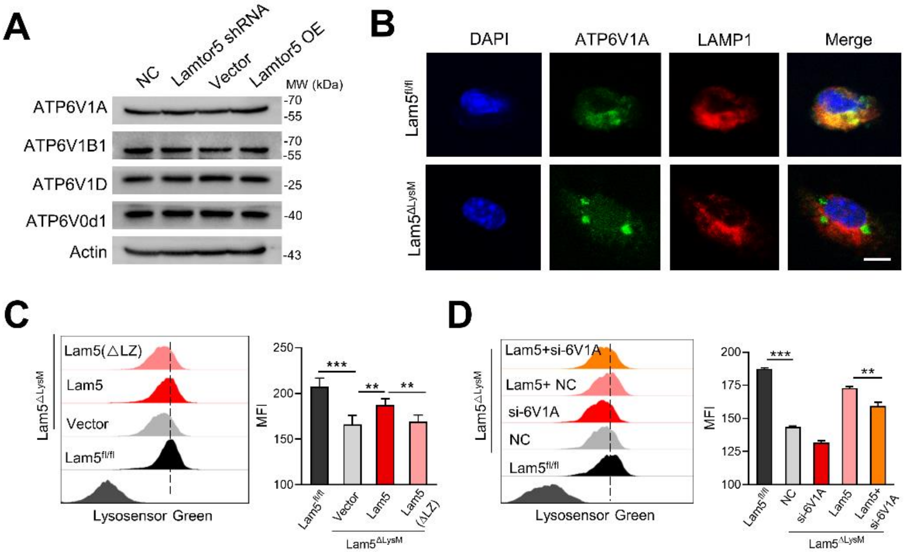

Figure S4. ATP6V1A/Lamtor5 interaction was required for v-ATPase activity in lysosomes. (A) The impact of Lamtor5 on the protein levels of different subunits of v-ATPase. Immunoblotting of ATP6V1A, ATP6V1B1, ATP6V1D and ATP6V0d1 in control, Lamtor5-expressing or Lamtor5-silenced RAW264.7 cells. β-actin was used as internal control. **(B)** Loss of Lamtor5 affected the binding of ATP6V1A to the LAMP1^{+} lysosome. Colocalization of ATP6V1A and LAMP1 in BMDMs from Lamtor5$^{\Delta LysM}$ and Lamtor5$^{fl/fl}$ mice was examined by confocal microscopy. Scale bar, 5μm. Shown are representative images from two or three independent experiments. **(C)** Replenishment of Lamtor5 on lysosomal acidification. Lamtor5 or Lamtor5(ΔLZ) was overexpressed in Lamtor5 deficient BMDMs (from 4-month-old mice), lysosomal acidification was determined by LysoSensor Green DND-189 staining followed by FACS analysis. Representative images and relative fluorescence quantification of LysoSensor Green are shown. **(D)** The effect of ATP6V1A knockdown on maintenance of lysosomal acidification. Lamtor5 and/or ATP6V1A siRNA were overexpressed in Lamtor5 deficient BMDMs (from 4-month-old mice). Lysosomal acidification was detected by Lysosensor Green DND-189 staining followed by FACS analysis.

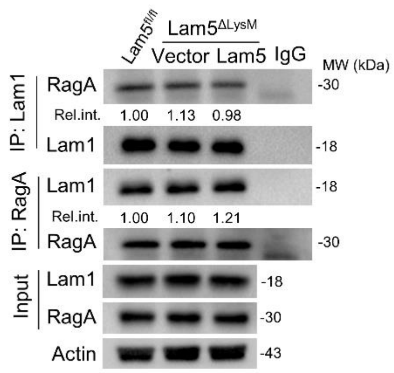

Figure S5. Lamtor5 has no effect on the interaction between Ragulator and RagA. Effect of Lamtor5 on the association of RagA and Lamtor1. Lamtor5 or vector was overexpressed in

Lamtor5 deficient BMDMs, the interaction of Lamtor1 and RagA was detected by co-immunoprecipitation. IgG was used as a control.

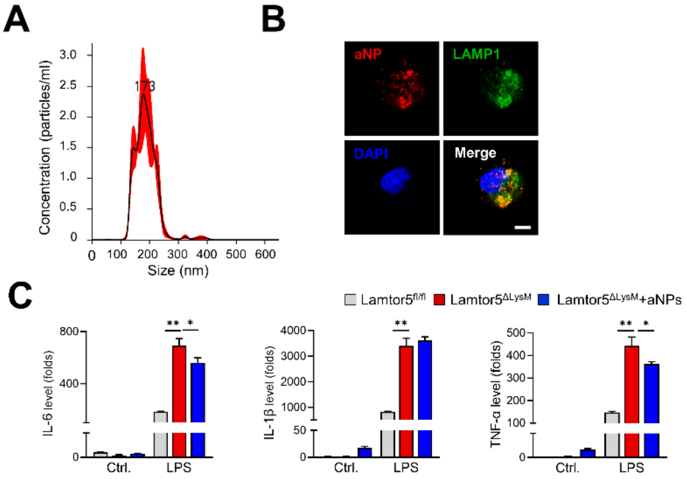

Figure S6. Particle size determination, lysosomal localization and anti-inflammatory effect of acidic nanoparticles. (A) Nanoparticle-tracking analysis of acidic nanoparticles (aNPs). aNPs (10 mg) were dissolved in ddH_2O (1 ml), sonicated at low frequency for 3 minutes and filtered, and then measured using a NanoSight NS300. Histogram showing the aNPs particle-size distribution. **(B)** Lysosomal localization of aNPs. RAW264.7 cells were treated with 3 mg/ml aNPs loaded with Nile red for 4 h. Colocalization of aNPs-Nile Red and LAMP1 were determined by Laser confocal microscope. Scale bar, 5 μm. **(C)** aNPs treatment inhibits the production of LPS-induced inflammatory factors in BMDMs. BMDMs from 4-month-old Lamtor5$^{\Delta LysM}$ and Lamtor5$^{fl/fl}$ mice were pretreated with or without 3 mg/ml aNPs for 4 h followed by LPS stimulation for 6 h. The production of IL-1β, IL-6 and TNF-α were detected by ELISA. The data from three independent experiments are expressed as means ± SD. *P < 0.05, **P < 0.01 by student's t test, as compared with non-stimulated controls.
